# Single-encounter elicitation framework for diagnostic excellence patient-reported measures: SEE-Dx-PRM

**DOI:** 10.1016/j.pecinn.2024.100357

**Published:** 2024-11-17

**Authors:** Vadim Dukhanin, Kathryn M. McDonald, Susan K. Peterson, Kelly T. Gleason

**Affiliations:** a Department of Health Policy and Management, Johns Hopkins Bloomberg School of Public Health, Baltimore, MD, USA; b Johns Hopkins University School of Nursing, Baltimore, MD, USA; c Armstrong Institute for Patient Safety and Quality, Johns Hopkins University School of Medicine, Baltimore, MD, USA; d Department of Emergency Medicine, Johns Hopkins University School of Medicine, Baltimore, MD, USA

**Keywords:** Diagnostic excellence, Patient-reported experience, Patient-reported outcome, Emergency care, Urgent care

## Abstract

**Objective:**

To create a conceptual framework for assessing patient-reported diagnostic excellence of a single diagnostic encounter.

**Methods:**

We held multiple expert panel meetings to prioritize a priori identified diagnostically relevant patient-reported experience and outcome domains. We combined and synthesized expert feedback with our experience in measure development and the reflections of a patient focus group. We then developed the framework, SEE-Dx-PRM (Single-Encounter Elicitation Framework for Diagnostic Excellence Patient-Reported Measures).

**Results:**

We defined the SEE-Dx-PRM's scope as intended for a single diagnostic encounter in emergency or urgent care, prospective and agnostic of the health condition, and with a timeframe of within several days up to a month from the encounter. The SEE-Dx-PRM's diagnostic excellence outcomes are: (1) accurate diagnosis and (2) either final, or working diagnosis, or specific next steps to establish diagnosis that were communicated and comprehended by patients. SEE-Dx-PRM encompasses 2 domains associated with accurate diagnosis, 5 domains of patient perception of iterative diagnostic process, 5 domains associated with communication and comprehension, and a domain associated with uncertainty.

**Conclusion:**

SEE-Dx-PRM-informed measures might support quality improvement, prompt system response, and research on diagnostic excellence.

**Innovation:**

SEE-Dx-PRM presents a novel patient-centered framework for the emerging diagnostic excellence construct and its measurement.

## Highlights

• Patient reporting is a powerful source to inform diagnostic excellence
• Developed framework – SEE-Dx-PRM – assesses such excellence of a single encounter
• SEE-Dx-PRM can inform quality improvement, prompt response actions, and research

## 1 Introduction

Diagnostic excellence is a nascent and evolving construct. Definitions of diagnostic excellence put forward to date explore a wide-ranging spectrum of diagnostic outcomes and aspects of the diagnostic process [1]. Patient-centeredness is inarguably a dimension of diagnostic excellence; this dimension emphasizes the importance and distinctiveness of the patient's perspective where a patient's understanding does not end with a diagnostic label but rather begins with one. It elaborates on the failure to communicate the explanation of the patient's health problem(s) to the patient, which constitutes a diagnostic error [2,3]. Patient-centeredness in diagnostic excellence does not seek to validate or contrast the patient's lived experience against clinical perspectives, but instead puts a unique value on experiences of those who are intimately involved.

Measurement of diagnostic excellence is also relatively new with its own challenges. It aims to identify best practices, provide positive feedback, and attribute diagnostic excellence in the context of contributions of the system versus team's (including the patient) versus individual clinician's [4,5]. When we examine diagnostic excellence within the context of a single diagnostic encounter, such as a visit to an emergency department or urgent care center, only some dimensions of diagnostic excellence are relevant. In single diagnostic encounters, there is often no prior established patient-clinician relationships nor relevant medical history. Single diagnostic encounters often lack any structured post-encounter outcome feedback to health care providers, and thus providers could benefit from collection of patient-reported information [6]. Patient input is particularly critical if a prompt response to that input could mitigate or prevent harms for that patient.

Patient reporting is increasingly recognized as a potentially powerful source for informing diagnostic excellence and can be particularly applicable to single diagnostic encounters [[7], [8], [9]]. Patient reporting captures the lived experiences of patients or their care partners, i.e., those who partner with patients to manage their care, such as family, friends, patient advocates, or other patient representatives. Those lived experiences can be described and systematized via domains of diagnostically relevant patient-reported experiences and outcomes [10]. However, there is not a comprehensive understanding of which patient-reported experience and outcome domains can inform diagnostic excellence of single diagnostic encounters, which domains are most relevant, and how these domains relate to each other.

Given this gap, we aimed to create a conceptual framework for assessing patient-reported diagnostic excellence of a single diagnostic encounter by structuring patient reports. This conceptualization entails describing the framework's scope, timeframe, intended use, and conceptual components including identified patient-reported domains and their relevance to diagnostic excellence.

## 2 Methods

This study builds on our initial work, in which we conducted a scoping review to identify diagnostically relevant patient-reported experience (PRE) and patient-reported outcome (PRO) domains [10]. The review included environmental scans of published and gray literature, a series of local expert consultations, additional environmental literature scans, the convening of a 24-member international expert panel, and the validation of the domains' diagnostic relevance via mapping these onto a set of patient diagnostic journeys. As a result of that published scoping review, 41 patient-reported experience and outcome domains were identified as building blocks for the emergent construct of diagnostic excellence and were inductively formed. The identified domains were agnostic of a symptom or health condition.

We then employed the Delphi method through multiple rounds of expert panel meetings to select the most relevant domains from the 41 identified [11]. The expert panel consisted of 9 members who were patients, patient advocates, physicians, healthcare administrators, and researchers in quality improvement, communication, biostatistics, diagnostic excellence, and patient-reported measure development. The experts were tasked with reviewing the identified domains and prioritizing them specifically in the context of a single diagnostic encounter, taking into account a timeframe for the measurement. For each domain, the experts were provided with examples from the literature of questions eliciting patient and care partner reporting on that domain. This resulted in a short-list of 26 domains and, with subsequent iterations, 13 final domains.

The research team's work proceeded in two directions. First, the team developed a new patient-reported measure, Patient-Report to IMprove Diagnostic Excellence in Emergency Department settings (PRIME-ED) [7,12,13], that consists of 17 items rated by patients and care partners on a 5-point Likert scale. In the second direction, the team used their expertise to theorize associations between the 13 domains and main outcomes of the diagnostic process. While theorizing [14,15], the research team did not aim to suggest causal relationships between the domains. This phase of work resulted in a draft framework with theorized associations.

To test face validity [16], we presented the draft framework to a 5-member focus group of patients and care partners who had had a recent single diagnostic encounter, specifically an emergency department or urgent care facility visit. The research team asked focus group members to reflect on how experiences and outcomes from that visit mapped onto the draft framework. This exercise helped to confirm the domains and their interrelations. This focus group also considered the scope. For instance, the focus group identified some factors that affect patient-reported excellence of a single diagnostic encounter, such as individual tolerance of uncertainty and medical insurance status. We then classified those factors as outside the scope of the framework.

Based on the focus group's input and the team's measure development experience, the conceptual framework was further refined, reorganizing theorized associations between the 13 domains into its final version, the Single-Encounter Elicitation Framework for Diagnostic Excellence Patient-Reported Measures (SEE-Dx-PRM).

## 3 Results

SEE-Dx-PRM consists of the following elements presented in sequence below: overarching scope, selected timeframe, intended use, diagnostic excellence outcomes, patient-reported experience and outcome domains, theorized associations between the domains, and the role of factors left outside the SEE-Dx-PRM's scope.

### 3.1 Framework scope

SEE-Dx-PRM conceptualizes both patients' and care partners' lived experiences regarding diagnostic excellence of a single diagnostic encounter with a clinical care team. We define a single diagnostic encounter as one for which a patient, clinician, or both have expectations that a new expressed health concern be diagnosed within this encounter, a conceptualization most suitable for emergency department and urgent care visits. Within this context, from the patients' perspective, diagnosis is seen as either final, working, or providing a set of specific next steps on the pathway to an explanation of the health concern, also known as safety-netting [17,19]. The framework scope of one encounter has an inherently prospective approach for capturing diagnostic excellence. The single encounter scope is also agnostic to the particular diagnosis, as it focuses on one opportunity to establish an explanation for any health concern and not on diagnosis or management of a specific condition.

### 3.2 Framework timeframe

What can be reported within a specific timeframe and how that timeframe of reporting could be useful are important considerations for collecting patient input. Self-reporting accuracy diminishes with the passage of time, so reporting within a short timeframe following the encounter would be desirable. However, allowing some time to pass post encounter gives a respondent the opportunity to integrate what happened within the visit and to report on how their health progressed afterward. Therefore, we conceptualize the SEE-Dx-PRM's timeframe as “moderately immediate,” or within several days up to a month. The goal of such a timeframe is to balance a respondent's immediate reflections on the encounter with those that are accumulated with enough time.

### 3.3 Framework intended use

The intended use of SEE-Dx-PRM is to highlight measurement opportunities for patient-reported diagnostic excellence of a single diagnostic encounter. Those measurement opportunities can be used to generate input for quality improvement and performance measurement, including identification of best practices and provision of positive feedback. Due to its prospective nature and relatively tight timeframe, patient-reported assessments additionally can be used to provide a prompt clinical response to specific care concerns identified via reporting. SEE-Dx-PRM also helps to inform an evolving construct of diagnostic excellence, its patient-centeredness, and other dimensions. Finally, SEE-Dx-PRM might direct future research and development of diagnostic excellence interventions.

### 3.4 The framework: Pictorial and written description

#### 3.4.1 The framework's diagnostic excellence outcomes (shown in turquoise)

The diagnostic excellence outcomes of the encounter are: (1) accurate diagnosis; (2) either established diagnosis that was communicated to and comprehended by the patient or, alternatively, if the diagnosis was not established, next steps to establish that diagnosis that are communicated to and comprehended by the patient; and (3) timely diagnosis.

The *accurate diagnosis* outcome was derived from the first part of the definition of the diagnostic error: the failure to establish an accurate explanation of the patient's health problem(s) [3]. The lack of a diagnostic error, a SEE-Dx-PRM's diagnostic excellence outcome, is an accurate diagnosis within the limitations of a single diagnostic encounter. Under those limitations, accurate diagnosis also includes no diagnosis when it is too early to establish the diagnosis accurately.

The *diagnosis communicated and comprehended by the patient* outcome was adapted from the second part of the definition of the diagnostic error: the failure to communicate the diagnosis to the patient [3]. Under SEE-Dx-PRM, the diagnosis should be received by the patient, understood by the patient, and that understanding then confirmed by the care team.

The *communication of next steps to establish the diagnosis* outcome was adapted from existing Consumer Assessment of Healthcare Providers and Systems (CAHPS) surveys, for instance, ED CAHPS used to assess emergency department visits [20,21]. As in CAHPS, under SEE-Dx-PRM, patients assess receiving follow-up instructions, yet in SEE-Dx-PRM patients also assess if they understand these next steps and if that understanding is confirmed by the care team.

The *timely diagnosis* outcome is embedded in the first part of the definition of the diagnostic error: the failure to establish a timely explanation of the patient's health problem(s) [3]. The SEE-Dx-PRM's timeframe and scope do not allow for patient or care partner's reporting of the timeliness of the diagnosis (this outcome's circle is grayed). The assessment of timeliness is done by retrospectively estimating the delay in establishing the diagnosis, while this framework is prospective. In addition, while timeliness is most often described as condition-dependent, the SEE-Dx-PRM is condition-agnostic [3].

#### 3.4.2 The framework's patient-reported experience and outcome domains (shown in dark blue)

The final 13 domains can be reported by both the patient and their care partner(s). Among those, two are associated with accurate diagnosis, five with patient perception of iterative diagnostic process, five with communication and comprehension, and one with uncertainty. One additional (fourteenth) domain, presence of diagnostic uncertainty, cannot be reported. These domains are presented below based on their relation to diagnostic excellence outcomes.

##### 3.4.2.1 Domains associated with accurate diagnosis (shown in the top left corner)

*Patient perception of subsequent health trajectory against the diagnosis* can be illustrated by questions in existing instruments. For example, a patient-reported outcome measure for use with adult ED patients who are discharged home, PROM-ED, asks patients to reflect on their recovering from the health condition that brought them to the diagnostic encounter and whether this recovery trajectory is anticipated based on the explanation of their health problem that they have received at that encounter [22]. In SEE-Dx-PRM, this domain includes situations when a health concern is undiagnosed and specific expectations of the health trajectory are unknown since further steps are required to establish the diagnosis.

*Patient perception and acceptance of diagnosis* is based on patients' reporting on their feelings, beliefs, or knowledge about the accuracy of the diagnosis as established at the encounter. Existing studies have demonstrated that patients can reliably report on the accuracy of their diagnoses. Such examples are the Patient-Reported Experiences and Outcomes of Safety in Primary Care (PREOS-PC) questionnaire and in computer-assisted telephone interviews [23,24].

##### 3.4.2.2 Domains of patient perception of iterative diagnostic process (shown along the light blue circular arrow)

*Relational* aspects of the iterative communication process are well-described. For instance, an integrative literature review illustrates and contextualizes relational aspects under patients' experiences of processes of communication [25,26]. Under SEE-Dx-PRM, the patient is assessing the extent to which they felt they were being treated as equal, being an important part of the team that establish the diagnosis, and whether they felt safe, comfortable, and accepted by the care team during the diagnostic encounter.

*Being listened to* and *taken seriously* domains are evaluated in existing CAHPS surveys, for instance, ED-CAHPS [21]. Patients are asked about whether they were treated with courtesy and respect and whether the care team listened carefully to the patient during the diagnostic encounter. These SEE-Dx-PRM's domains also encompass patient reflections on the length of the encounter needed to be heard and patient reflections on the actions of the care team, such as documenting patient's concerns or asking for further details, demonstrating that patient's concerns are taken seriously.

*Patient understanding of diagnostic process* is a reflection on what healthcare providers share with a patient about their actions or potential actions during the encounter. As part of this SEE-Dx-PRM's domain, patient understanding also relates to the extent of meaningful shared decision making, shared mental model, and diagnostic co-production [27,28].

*Interpersonal adaptation* is described in relation to communication dynamics. For example, Hannawa describes this domain as “the extent to which participants respond to implicitly (i.e., nonverbally) and explicitly (i.e., verbally) expressed needs and expectations to maximize the likelihood of shared understanding” in the SACCIA Safe Communication (i.e., part of five core competencies for safe and high-quality care) [26]. Another example can be found in a qualitative study of patients' views on clinician initial communication about lung cancer screening [29]. Within SEE-Dx-PRM, this domain includes how well a patient's specific needs and expectations, whether the same or different from other patients, were understood and adapted to by the care team.

##### 3.4.2.3 Domains associated with communication and comprehension of diagnosis or of next steps to establish the diagnosis (shown in dark blue in the framework's lower part)

*Clear communication* is defined in the SACCIA Safe Communication [26] as “the extent to which participants express and interpret verbal and nonverbal messages clearly (i.e., unambiguously) and utilize their interaction with each other to reduce uncertainty.” Under SEE-Dx-PRM, the patient assesses the extent of clarity of information, oral and written, received from members of the care team during a single diagnostic encounter. It includes reflection on the perceived clarity of information that might contain medical jargon or specialized language.

*Sufficient communication*, another part of SACCIA Safe Communication, is defined as “the extent to which participants convey, extract, and exchange a sufficient amount of information in order to arrive at a shared understanding” [26]. Under SEE-Dx-PRM, the patient assesses the extent of sufficiency of communication of diagnosis or communication of next steps to establish the diagnosis. The impact that shared understanding should achieve is described in the next domain.

*Functionality of information* so that communication conveys a plan to follow is present in several questions of PROM-ED [22]. This domain of “having a plan to follow” is described as: “[m]any issues were not completely resolved during the course of the ED visit, and patients highlighted the need for a plan as a valued outcome of the ED visit.” Under SEE-Dx-PRM, the patient assesses whether the information received enabled the patient to manage the diagnosed health problem. For example, whether they know what to watch for, what doctors they need to follow up with for treatment, or whether they know what to do if the health problem(s) gets worse.

*Patient's comprehension of the diagnosis* is captured by validated instruments, such as ED CAHPS, where patients assess whether providers explained things in a way that patients could understand [21]. Under SEE-Dx-PRM's domain, patients can be asked directly if they have complete comprehension of their diagnosis or the next steps to establish of the diagnosis. Beyond capturing the extent to which information is sufficient, clear, or functional, this domain assesses the overall understanding of the diagnosis.

*Providers' checking of patient understanding* assesses the presence of the process of checking patient understanding as a part of the encounter rather than the understanding itself [30]. Patients assess whether this process has taken place during their encounter.

##### 3.4.2.4 Domains associated with diagnostic uncertainty (shown in the middle, to the left from the light blue circular arrow)

The *presence of diagnostic uncertainty* is an important domain, yet it cannot be assessed by patient reporting. The presence of uncertainty might manifest as either the inability of the care team to establish the diagnosis within one diagnostic encounter or as a degree of uncertainty surrounding the established diagnosis itself. From the patient reporting perspective, what can be captured is the *communication of uncertainty* to a patient. This SEE-Dx PRM's domain tackles communicating uncertainty with clarity and sufficiency so that the patient comprehends the uncertainty, and so that providers check that understanding with the patient. When the uncertainty does not allow establishing the diagnosis, under SEE-Dx-PRM, the patient assesses the communication and comprehension of next steps needed to establish the diagnosis with higher certainty.

### 3.5 Associations between framework's domains (shown as blue arrows and blue shading)

**Fig. 1:**
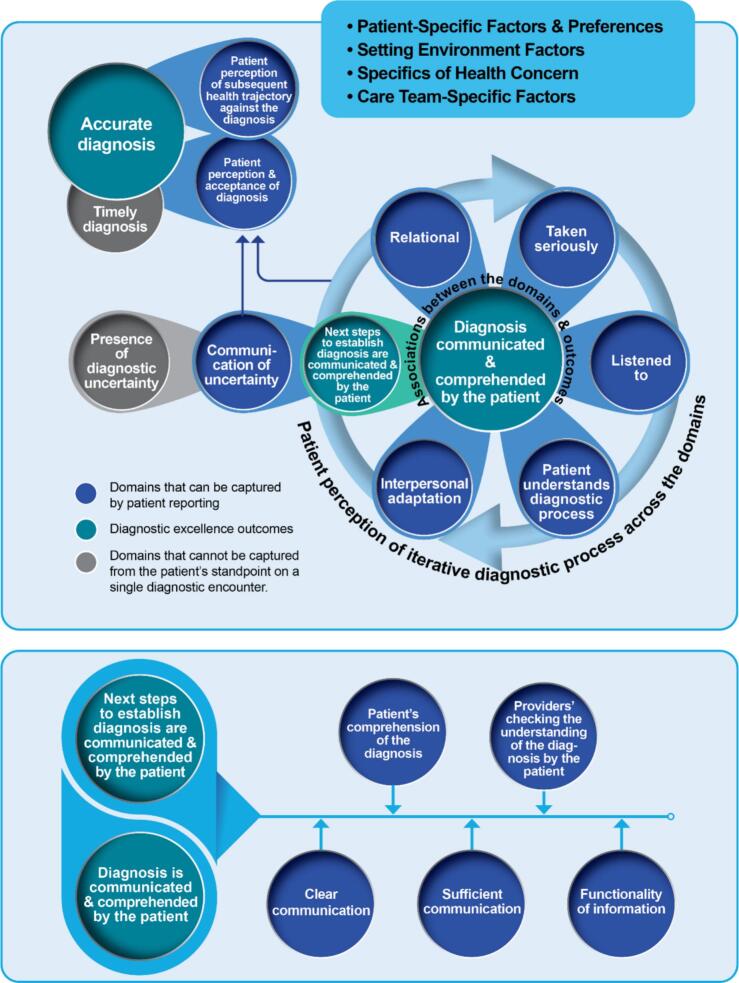
Single-Encounter Elicitation Framework for Diagnostic Excellence Patient-Reported Measures (SEE-Dx-PRM). Dark blue circles denote *domains* that can be captured by patient reporting (the final 13 domains that include those in the framework's lower part), while gray ones represent domains that cannot be captured from the patient's standpoint on a single diagnostic encounter. Turquoise circles show diagnostic excellence *outcomes* that can be captured by patient reporting through associated domains; two of the outcomes are repeated in the framework's lower part to show additional domains and associations. The outcome in gray cannot be captured from the patient's standpoint on a single diagnostic encounter. Blue *arrows* and blue *shading* show associations between the domains and outcomes. Light blue *circular arrow* represents that the patient perception of iterative diagnostic process builds across the domains. The blue rectangular *border* separates SEE-Dx-PRM from four sets of factors (listed in the top right box) that influence diagnostic excellence but are beyond the framework's scope of patient reporting.

As presented in the Fig. 1, the 13 SEE-Dx-PRM's domains are conceptualized as primarily associated with specific diagnostic excellence outcomes. Two domains (patient perception of subsequent health trajectory against the diagnosis and patient perception and acceptance of diagnosis) are associated with accurate diagnosis. Five domains (relational, listened to, taken seriously, patient understanding of diagnostic process, and interpersonal adaptation) are domains that form patient perception of diagnostic process. Together, they are associated with communication and comprehension by the patient of the diagnosis or the next steps to establish the diagnosis. Additionally, the domains of patient perception of diagnostic process and of diagnosis (and its acceptance) are associated. Another five domains (clear communication, sufficient communication, functionality of communication, patient comprehension, and providers' checking of patient understanding) are associated with communication of diagnosis or next steps to establish the diagnosis. The final domain, the ability to communicate the uncertainty, is associated with whether the patient comprehends the diagnosis or understands that the uncertainty requires additional next steps to establish the diagnosis. Communication of uncertainty is also associated with patient perception and acceptance of diagnosis.

### 3.6 The framework's sets of factors beyond the scope of patient-reported measurement (shown in the top right corner box)

The SEE-Dx-PRM conceptualizes four sets of largely distinct factors, depicted as surrounding a single diagnostic encounter, that affect patient-reported excellence of a diagnostic encounter. They are: patient-specific factors and preferences; setting environment factors; specifics of health concern; and care team-specific factors (see Appendix). Although they may be pertinent for risk adjustment or valuable for choosing among diagnostic performance improvement options, these four sets are not measured under SEE-Dx-PRM via patient reporting.

## 4 Discussion and conclusion

### 4.1 Discussion

We present a conceptual framework, SEE-Dx-PRM, created to identify and describe domains of patient-reported diagnostic excellence and their associations within the context of a single encounter. SEE-Dx-PRM describes measurement opportunities for diagnostic excellence in a timeframe proximal to a single diagnostic encounter (i.e., moderately immediate timeframe) for patient or care partner reporting. Given the anticipated value of patient reports to guide diagnostic care quality improvement efforts, SEE-Dx-PRM may be used to guide the development of patient-reported instruments in the context of single-encounter visits, such as emergency department or urgent care visits.

Patient-reported diagnostic excellence aims to complement an evaluation of diagnostic excellence from healthcare provider and system standpoints. A cumulative investigation from multiple sources can provide more nuanced targets for feasible improvements [7,31,32]. In SEE-Dx-PRM, reflecting a patient lens, only domains that require the patient view are included. Aspects such as setting environment and care team characteristics and processes, shown on its edge, are considered outside of SEE-Dx-PRM's scope. This contrasts or complements other frameworks that employ differing lenses [3,33].

SEE-Dx-PRM posits the construct of patient-reported diagnostic excellence of a single diagnostic encounter separate from patient satisfaction. By decomposing this construct on distinct assessable components and highlighting associations, SEE-Dx-PRM identifies targets for local quality improvement, prompt response to specific concerns, and future research. As the patient-reported diagnostic excellence construct matures, current terminology choices drawn from the field of diagnostic quality (e.g., diagnostic process, uncertainty, and timeliness) may be further elaborated or replaced to assure optimal data capture. As such data collection can be done via patient-reported measures, patient narratives, or other novel methods, nuances of terminology (e.g., diagnostic problems and mistakes instead of errors [8,9]) will continue to emerge and allow the evolution of SEE-Dx-PRM.

#### 4.1.1 Limitations

By focusing on one diagnostic encounter, SEE-Dx-PRM does not consider patient-reported measurement opportunities related to: (1) entry into the care, e.g., barriers to access; (2) outcomes of the treatment plan developed as a result of the established and communicated diagnosis; or (3) connectedness and coordination between several diagnostic encounters. Our framework, agnostic of diagnosis or condition, might have taken an alternative approach by focusing on specific, pre-determined urgent health conditions that are typically diagnosed within one encounter. While more detail on a pre-defined subset of patients with particular diagnoses would potentially enhance applicability to such subgroups, it would introduce biases associated with retrospective selection. One other limitation of the diagnosis-agnostic choice is an inability to assess timeliness, one of the six dimensions of diagnostic excellence, that is generally described as condition-dependent and measured retroactively [1].

SEE-Dx-PRM's timeframe does not allow the inclusion of patient reflections on the diagnostic encounter over a longer period. Information is missed due to the shorter timeframe. For example, the reflections do not cover how the patient may feel after treatment for a diagnosed condition, or if a long-lasting diagnostic experience affected future healthcare-seeking behavior. Even in a shorter term, as a patient and their care partner venture through an iterative diagnostic process that might have a non-linear trajectory over time, the patient's expectations change, lived experience with a new health condition matures, perception and acceptance of the diagnosis evolve, and interactions with health systems and providers accumulate [34].

SEE-Dx-PRM implicitly allows for conceptualizing measurement opportunities to detect diagnostic disparities that reflect received inequitable care. These include assessing disparities occurring due to: (1) vulnerabilities in experiences of the communication during the diagnostic process; (2) different salience and importance of SEE-Dx-PRM domains for populations with vulnerabilities; (3) potentially greater power differential with care team members that might influence how vulnerable patients report; and (4) response or non-response rates among those vulnerable. The vulnerability factors can include salient and visible factors such as age, race or ethnicity, gender identity, English language proficiency, health literacy level, housing instability, and others [35]. However, given SEE-Dx-PRM's scope, diagnostic disparities that occur due to different experiences with entrance to or engagement with health systems or different use of healthcare settings are not reflected.

#### 4.1.2 Future directions

Future directions include extending SEE-Dx-PRM to different timeframes, for example, considering the implications for measuring in two stages: first gathering reflections on encounter as close to the encounter as feasible and then seeking reflections on the encounter's outcomes at another, more distant time point. Another direction would be to explore the domain of uncertainty more extensively, given the growing attention for achieving this aspect of diagnostic excellence where clinicians' and patients' perspectives are distinct [36,37]. One possibility would be to explore and decompose the assessment of communication of uncertainty and its relation to measures of the presence of diagnostic uncertainty, the latter most likely not to be patient-reported. Finally, it is worth investigating whether SEE-Dx-PRM can be used in the context of patient-reported measurement of diagnostic excellence of primary care visits where a patient presents with a new health concern.

### 4.2 Innovation

SEE-Dx-PRM is novel in its exploration of the nascent diagnostic excellence construct, where it examines the patient-centeredness dimension of diagnostic excellence through the lens of patient reporting. Furthermore, SEE-Dx-PRM introduces a set of concrete domains to be assessed and theorized associations between those domains that will guide future measure development. This has potential to significantly contribute to a growing area of diagnostic excellence measurement centering on what matters to patients and their care partners.

### 4.3 Conclusion

We present a conceptual framework for assessing patient-reported diagnostic excellence of a single diagnostic encounter, SEE-Dx-PRM. This framework, with its prospective and condition-agnostic scope, moderately immediate timeframe, and identified domains and their associations, establishes a conceptual and practical base for patient-centered measurement development that can inform quality improvement and prompt response actions. SEE-Dx-PRM can be used to undergird future research on and the evolution of the emerging diagnostic excellence construct.

## CRediT authorship contribution statement

**Vadim Dukhanin:** Writing – review & editing, Writing – original draft, Formal analysis, Data curation, Conceptualization. **Kathryn M. McDonald:** Writing – review & editing, Writing – original draft, Methodology, Conceptualization. **Susan K. Peterson:** Writing – review & editing, Writing – original draft. **Kelly T. Gleason:** Writing – review & editing, Writing – original draft, Supervision, Methodology, Funding acquisition, Formal analysis, Conceptualization.

## Declaration of competing interest

Vadim Dukhanin reports financial support was provided by Gordon and Betty Moore Foundation. Kathryn McDonald reports financial support was provided by Gordon and Betty Moore Foundation. Susan Peterson reports financial support was provided by Gordon and Betty Moore Foundation. Kelly Gleason reports financial support was provided by Gordon and Betty Moore Foundation.

## Appendix A Supplementary data

The framework’s four sets of factors beyond the scope of patient-reported measurement Image 1
