# Supplementary material for: Single-encounter elicitation framework for diagnostic excellence patient-reported measures: SEE-Dx-PRM
Source: PEC Innov. 2024 Nov 17;5:100357. doi: 10.1016/j.pecinn.2024.100357 (PMC11617112; doi:10.1016/j.pecinn.2024.100357)
Supplement: Supplementary file 1 — The framework’s four sets of factors beyond the scope of patient-reported measurement [file mmc1.docx]

Single-Encounter Elicitation Framework for Diagnostic Excellence Patient-Reported Measures: SEE-Dx-PRM

**Appendix**

**Patient-specific factors and preferences** encompasses many personal characteristics, such as:

- patients’ own communication abilities and preferences for communication and information;
- patients’ preference for own level of involvement in diagnostic process (from being a co-partner in diagnosis production to the paternalistic model);
- patients’ preference for the level of transparency and disclosure of their health and other information;
- patients’ a priori beliefs and attitudes towards their health, healthcare systems, and care team members;
- patients’ care seeking behavior;
- patients’ perception of the power differential between them and care team members;
- patients’ prior experiences and pre-encounter expectations;
- patients’ understanding of the overall diagnostic process, tolerance of uncertainty and risks.

Those personal characteristics can be also reflected in other composite factors, such as patient satisfaction with the encounter or expectations for the encounter. The patient specific factors also encompass patients’ socio-economic status that would affect, for example, patients’ access to alternative healthcare providers or resources needed to follow through the diagnostic plan established at the encounter or patients’ other specific non-health capabilities and needs.

**Setting environment factors**: the framework’s scope limits applicable clinical setting instances to those that establish diagnoses within a single diagnostic encounter. Relevant factors will be setting diagnostic capacities, their access to patient’s health records, and how routinely the setting is accustomed to establishing diagnoses within a single diagnostic encounter. Another factor is patient waiting time of a diagnostic encounter, which will impact the patient’s overall experience.

**Specifics of health concern**: relate to the urgency and acuteness of a condition(s) the patient requires a diagnosis for, other concomitant health condition(s) and overall healthcare utilization and health status, including patients’ cognitive abilities at the time.

**Care team-specific factors** are the size and the composition of the care team, and individual members’ diagnostic and communication skills, prior experiences, preferences for patient and care partners’ roles in the diagnostic process, and care team members’ tolerance of uncertainty and risks, and ultimately responsiveness to and management of patient expectations. The written communications of the diagnosis from the team, however, will be influenced by additional stakeholders in the healthcare institutions. Organizational policies, practices, including incentives, will similarly impact the care team’s approach to diagnostic process as well. Thus, setting environment also impacts care team-specific factors.
